# Identification of miRNAs Potentially Involved in Bronchiolitis Obliterans Syndrome: A Computational Study

**DOI:** 10.1371/journal.pone.0161771

**Published:** 2016-08-26

**Authors:** Stefano Di Carlo, Elena Rossi, Gianfranco Politano, Simona Inghilleri, Patrizia Morbini, Fiorella Calabrese, Alfredo Benso, Alessandro Savino, Emanuela Cova, Davide Zampieri, Federica Meloni

**Affiliations:** 1 Control and Computer Engineer Department, Politecnico di Torino, Torino, Italy; 2 Department of Molecular Medicine, University of Pavia, Pavia, Italy; 3 Department of Internal Medicine, University of Pavia, Pavia, Italy; 4 Department of Respiratory Diseases IRCCS Policlinico S. Matteo, Pavia, Italy; 5 Department of Cardiovascular and Thoracic Sciences, University of Padova, Padova, Italy; 6 Thoracic Surgery Unit, Department Cardiothoracic and Vascular Sciences, University of Padova, Medical School, Padova, Italy; National Institute of Technology Rourkela, INDIA

## Abstract

The pathogenesis of Bronchiolitis Obliterans Syndrome (BOS), the main clinical phenotype of chronic lung allograft dysfunction, is poorly understood. Recent studies suggest that epigenetic regulation of microRNAs might play a role in its development. In this paper we present the application of a complex computational pipeline to perform enrichment analysis of miRNAs in pathways applied to the study of BOS. The analysis considered the full set of miRNAs annotated in miRBase (version 21), and applied a sequence of filtering approaches and statistical analyses to reduce this set and to score the candidate miRNAs according to their potential involvement in BOS development. Dysregulation of two of the selected candidate miRNAs–*miR-34a* and *miR-21 –*was clearly shown in in-situ hybridization (ISH) on five explanted human BOS lungs and on a rat model of acute and chronic lung rejection, thus definitely identifying *miR-34a* and *miR-21* as pathogenic factors in BOS and confirming the effectiveness of the computational pipeline.

## Introduction

Since James Hardy performed the first human lung transplantation (LTx) in 1963, LTx has become an accepted therapeutic option for carefully selected patients with an end-stage lung disease. The number of LTx has increased steadily over the last two decades and currently more than 3,000 lung transplantation procedures are performed yearly worldwide. LTx mainly intends to improve survival. However, long-term survival is hampered by the development of chronic lung allograft dysfunction (occurring in up to 50% of patients in the 5th post-transplant year), whose main clinical manifestation is represented by Bronchiolitis Obliterans Syndrome (BOS) [1]. As a consequence, long-term survival after LTx remains worse than that achieved for other solid organ transplantations (median overall adult survival, 5.6 years according to the latest ISHLT figures https://www.ishlt.org/registries/quarterlyDataReport.asp).

Histologically, BOS is characterized by Bronchiolitis Obliterans (BO), i.e. patchy submucosal fibrosis involving the respiratory bronchioles, resulting in near-total or total occlusion of the airway lumen. BOS is the final outcome of an array of injuries to the airway epithelium and extracellular matrix including alloimmune-specific reactions, autoimmune responses and non-specific inflammatory insults (infections and gastro-esophageal reflux). The exact pathogenesis of BOS is still largely unknown. It is believed that an insult to the epithelium leads to an inflammatory response that might be associated with a shift from tolerance to immune activation, which in turn induces severe neutrophilic airway inflammation [2]. Activated neutrophils can further damage the epithelium by releasing reactive oxygen species, chemokines, alarmins and metalloproteinases (MMPs). This inflammatory phase is ultimately followed by a fibro-proliferative phase leading to transition from epithelial to mesenchymal cells, proliferation of myofibrocytes, and collagen deposition. Even if the pathogenesis of BOS following LTx has not been clarified completely, recent studies suggest that epigenetic regulations involving microRNAs may play an important role in its development [3][4][5].

MicroRNAs (miRNAs) are the most studied class of small non-coding RNAs, containing approximately 20 nucleotides. They regulate post transcriptional gene expression by binding to target messenger RNAs (mRNAs) and inducing either inhibition of translation or mRNA degradation [6][7][8][9]. It is thought that miRNAs regulate approximately 30% of the human protein-coding genome [6]. They control the expression of genes involved in several key biological processes such as cell development, stem cell proliferation, division and differentiation, regulation of immunity, apoptosis, cell signaling and metabolism, etc. [6][8][9][10][11]. Moreover, aberrant expression of miRNAs has been associated with several pathological processes, such as cancer [9][12][13], metabolic disorders [14], autoimmune diseases [11] and acute cellular rejection following kidney transplantation [15]. Clarifying the role of miRNAs in disease pathogenesis is therefore crucial.

There are two approaches to the study of the role of miRNAs in diseases. The traditional "omics" approach assesses a wide panel of factors on plasma/tissue samples and identifies those significantly (2-fold to 10-fold) dysregulated in disease samples with respect to controls. In the other, computational algorithms are used to perform a preliminary exploratory analysis to narrow down number of factors to test in the subsequent wet-lab validation phase. These approaches, however, do not always provide information on the specific cellular site of expression, which is far more relevant than absolute expression levels.

In this paper, we implemented a pure computational pipeline to perform enrichment analysis of miRNAs in pathways involved in BOS pathogenesis. This enabled us to identify a limited panel of candidate miRNAs with potential involvement in BOS development. Unlike in other approaches, such as GSEA [16] and miTEA [17], we did not rely on expression profiles data to perform our analysis, but instead we integrated and processed information from a large set of different repositories, thus performing a pure exploratory analysis to pinpoint which specific wet-lab experiments would be most useful.

Preliminary validation of the selection of miRNAs was attained both through literature analysis during the computational phase; and by subsequent in situ hybridization experiments on *miR-34a* and *miR-21*, two of the resulting candidate miRNAs. Interestingly, hybridization experiments clearly showed, for the first time, dysregulation of these two miRNAs at cellular level in BOS tissue samples. This provided important insights into their role in the pathogenesis of BOS, opening perspectives for further mechanistic and therapeutic studies.

## Materials and Methods

**Fig 1:**
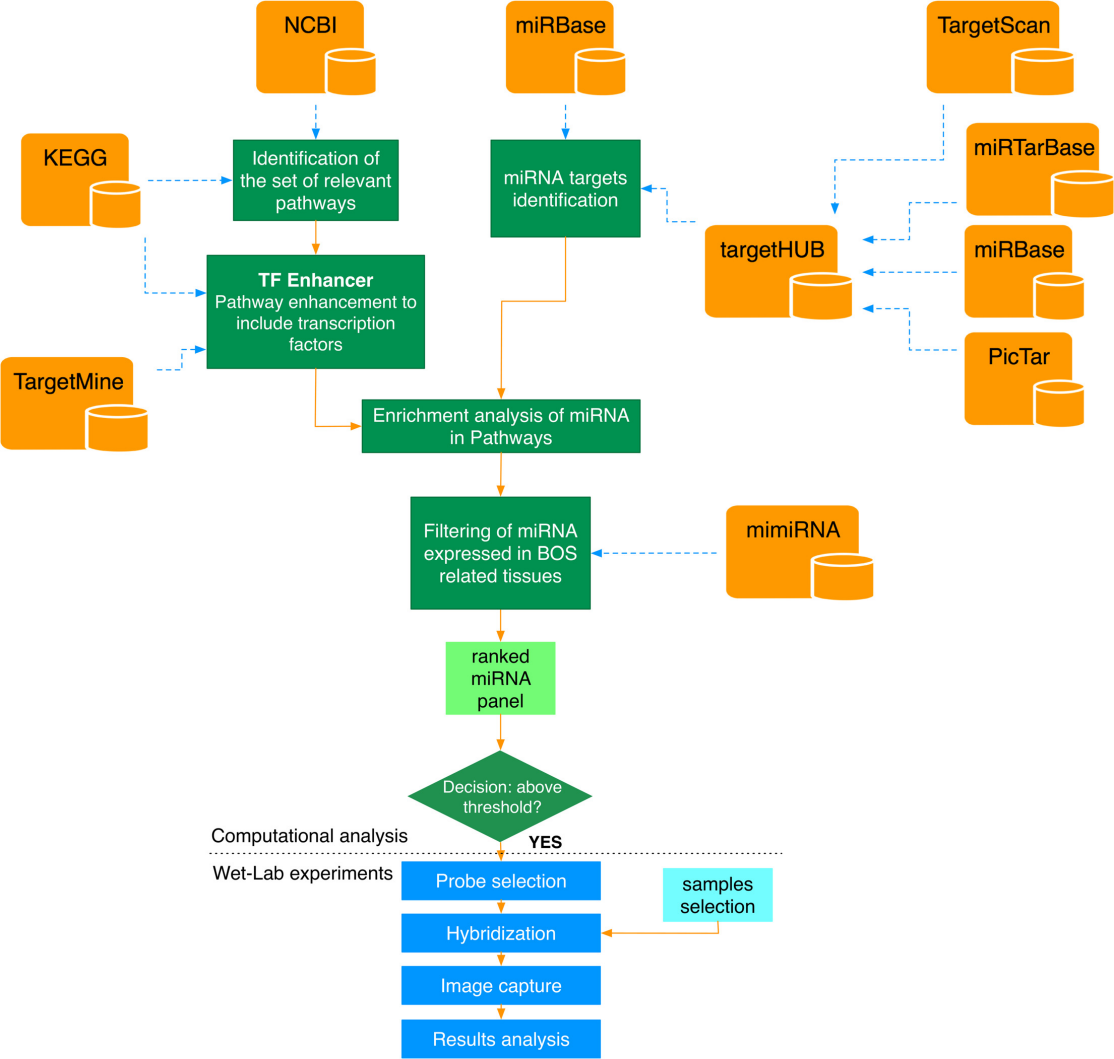
Computational workflow followed to identify relevant miRNAs (green and orange blocks). Two selected miRNAs (i.e., *miR-34a* and *miR-21*) obtained from the computational analysis were then validated in wet lab experiments (blue boxes).

Fig 1 shows the experimental workflow followed in this study. Green boxes identify the computational pipeline implemented to identify the candidate miRNAs involved in BOS, whereas blue boxes identify wet-lab activities performed for validation of the results. The computational pipeline implements a set of data fusion engines able to automatically collect and integrate omics information from a set of public repositories (orange boxes in Fig 1). Collected data are then analyzed through a set of statistical tests to score the analyzed miRNAs. The source code and the related data implementing the computation pipeline described in Fig 1 are publicly available through the BOS-miRNA-Enrichment repository on GitHub at https://github.com/sysbio-polito/BOS-miRNA-Enrichment.

### Identification of the set of relevant pathways

**Fig 2:**
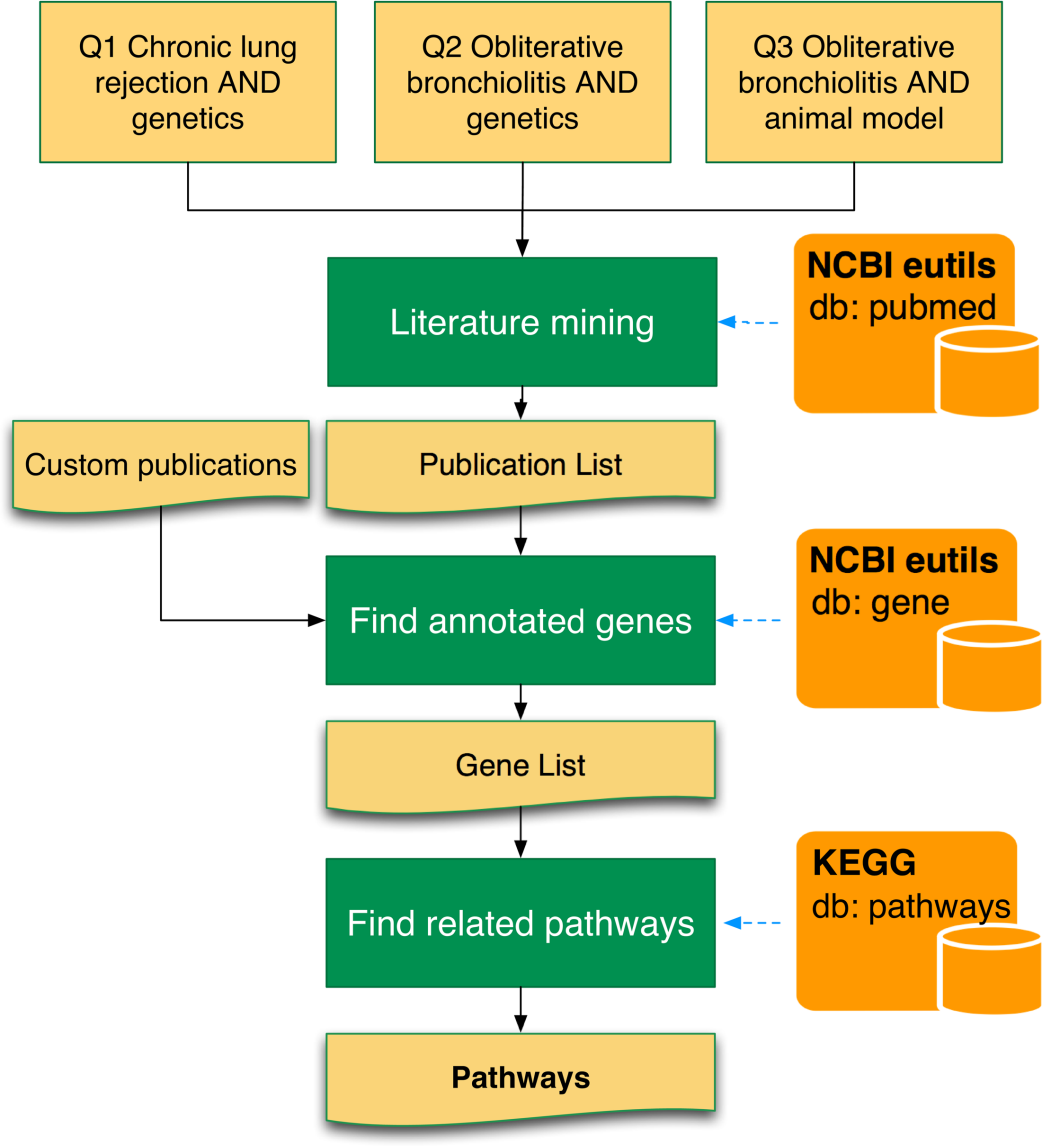
Pathway selection process. The NCBI E-Utilities Application Programming Interface (API) and KEGG API were used to mine the available literature and to identify a preliminary set of relevant pathways connected to BOS pathogenesis.

The proposed computational workflow starts from the identification of a set of relevant pathways behind key biological processes involved in the development of BOS. Several pathway repositories collect curated information on a large number of biological networks. These can be explored and analyzed for high-level systemic analysis [18, 19]. In this paper we resorted to the list of networks available in the KEGG database (http://www.genome.jp/kegg/pathway.html). KEGG is unique in its focus on and coverage of yeast, mouse, and human metabolic and signaling pathways, with a set of more than 200 networks for *Homo sapiens* [20]. To support the pathways identification process, we developed the automatic literature mining workflow, reported in Fig 2, whose code is available in the “1-literature-mining” folder of the BOS-miRNA-Enrichment repository on GitHub.

The workflow starts with a search of the available literature using NCBI E-utilities [21], with the three queries (Q1-Q3) reported in Fig 2. Each query contained associations of key terms related to BOS enabling the retrieval of a collection of relevant publications. This set of publications was further refined by including custom studies not directly identified by the three queries but known to be relevant to BOS. We used the NCBI E-utilities again on the new list to identify related genes annotated in the selected publications. Finally, starting from the list of genes identified, we used KEGG API to search for KEGG pathways annotated for the identified genes.

The automatic literature mining process identified a list of 51 pathways (see the “Results” folder of the BOS-miRNA-Enrichment repository on GitHub) that were then manually reviewed and filtered. This step was important to remove false positive pathways that might have been introduced by the automatic literature mining process or to add pathways eluded in the automatic search. This resulted in the list of 39 pathways reported in the S1 Table. Each pathway was assigned to one out of three classes, depending on their assumed relevance in the pathogenesis of BOS. Class A identified highly relevant pathways behind very specific biological processes related to the pathogenesis of BOS. Class B identified pathways relevant in more general processes compared to class A, but still important in the development of the disease. Finally, class C pathways are very general pathways with reduced specificity in the development of BOS. Assignment to the different classes and review of the pathways list was carried out by three researchers expert in BOS pathogenesis: a clinician (FM), a pathologist (PM) and a genetist (ER). This information was necessary to weigh the contribution of any given pathway during computational analysis, in order to avoid bias due to low specificity of pathways within the targeted pathology.

### Pathway extension to include transcription factors

Starting from the identified set of relevant pathways for BOS development, our computational workflow was used to search for possible interactions between miRNAs and genes involved in the different pathways. While direct interactions between miRNAs and their target genes can be retrieved from publicly available repositories, as explained in the sections that follow, it is well known that miRNA-mediated post-transcriptional regulation of selected genes can also act indirectly by translational repression or mRNA degradation of their transcription factors (TFs) [10][22][23][24]. Considering these indirect interactions is therefore very important when analyzing miRNA-pathway interactions. However, pathway repositories, such as KEGG, usually lack systematic information regarding the TF of each gene involved in a given regulatory network.

To take into account TF-mediated miRNA regulations, we developed a php script able to process the KEGG Markup Language (KGML) description of a pathway and systematically integrate TF information for each described gene (see Fig 1). TF information is retrieved by connecting to the RESTful APIs provided by TargetMine [25]. For a given gene, the “Upstream Transcription Factors Template Query” (Targetmine) enables the retrieval of all upstream regulatory genes (TFs) from the AMADEUS [26][27] and ORegAnno [28] libraries of transcription factors-target gene relations. By applying this computational step, each pathway considered was extended to systematically include the TFs for all the genes involved. The source code used to process each pathway is available in the “2-Pathway-Processing” folder of the BOS-miRNA-Enrichment repository on GitHub.

### Identification of miRNA

Our computational selection of candidate miRNAs involved in BOS development started from the full list of miRNAs annotated in miRBase (version 21 published on June 2014) [29]. MiRBase is a searchable database of published miRNA sequences. Version 21 of miRBase was exported in its SQL format and a list of 1870 miRNA precursor sequences for *Homo sapiens* species representing our initial list of miRNAs was extracted.

In order to perform the miRNA target enrichment analysis, we first retrieved miRNA target information for each miRNA contained in our initial list. miRNA target information was retrieved by automatically mining the TargetHUB repository with a php script (see Fig 1). TargetHUB provides a programmer-friendly interface to aggregate multiple repositories of miRNA target genes with a uniform set of APIs [30]. The TargetHUB interface allows users to query information from four different databases: miRTarBase [31], TargetScan [32], PicTar [33], and miRanda [34]. TargetHUB uses miRBase Version 18 nomenclature. Because the nomenclature for miRNAs is not completely standard across different versions of miRBase, names of those miRNAs whose names in miRBase (version 21) match miRBase (version 18) remained unchanged. miRNAs that had no matching identifier were manually mapped resorting to the name change logs available in miRBase.

In our study, we analyzed each miRNA considering two different sets of miRNA targets:

- Targets obtained by querying TargetHUB with results collected from the miRTarBase database referred to as Validated miRNA Targets (VMTs). MiRTarBase is known to collect information related to experimentally validated miRNA targets [35].
- Targets obtained by querying TargetHUB for results present in at least one of the five available repositories. These are referred to as Computational miRNA Targets (CMTs) since they contain a large set of computationally-inferred information.

VMTs were the main focus of this research and were used as the main source of information to identify the set of miRNAs to be tested in the laboratory. Nevertheless, CMTs were also of interest because they enlarged the scope of the analysis and allowed the formulation of exploratory predictions on miRNAs, to be tested in future experiments. In the remainder of this paper we will use the general term miRNA targets to identify both VMTs and CMTs. The set of scripts used to perform this computational step is available in the “3-Mirna-Processing” folder of the BOS-miRNA-Enrichment repository on GitHub.

### miRNAs enrichment analysis

To identify relevant miRNAs in BOS development we performed enrichment analysis of miRNA gene targets in the selected pathways for each of the 1870 considered miRNAs. This analysis allowed us to rank miRNAs based on their overall enrichment in the selected pathways. This information was used to skim the initial list of miRNAs, identifying a reduced panel with potential higher involvement in the pathogenesis of BOS.

**Fig 3:**
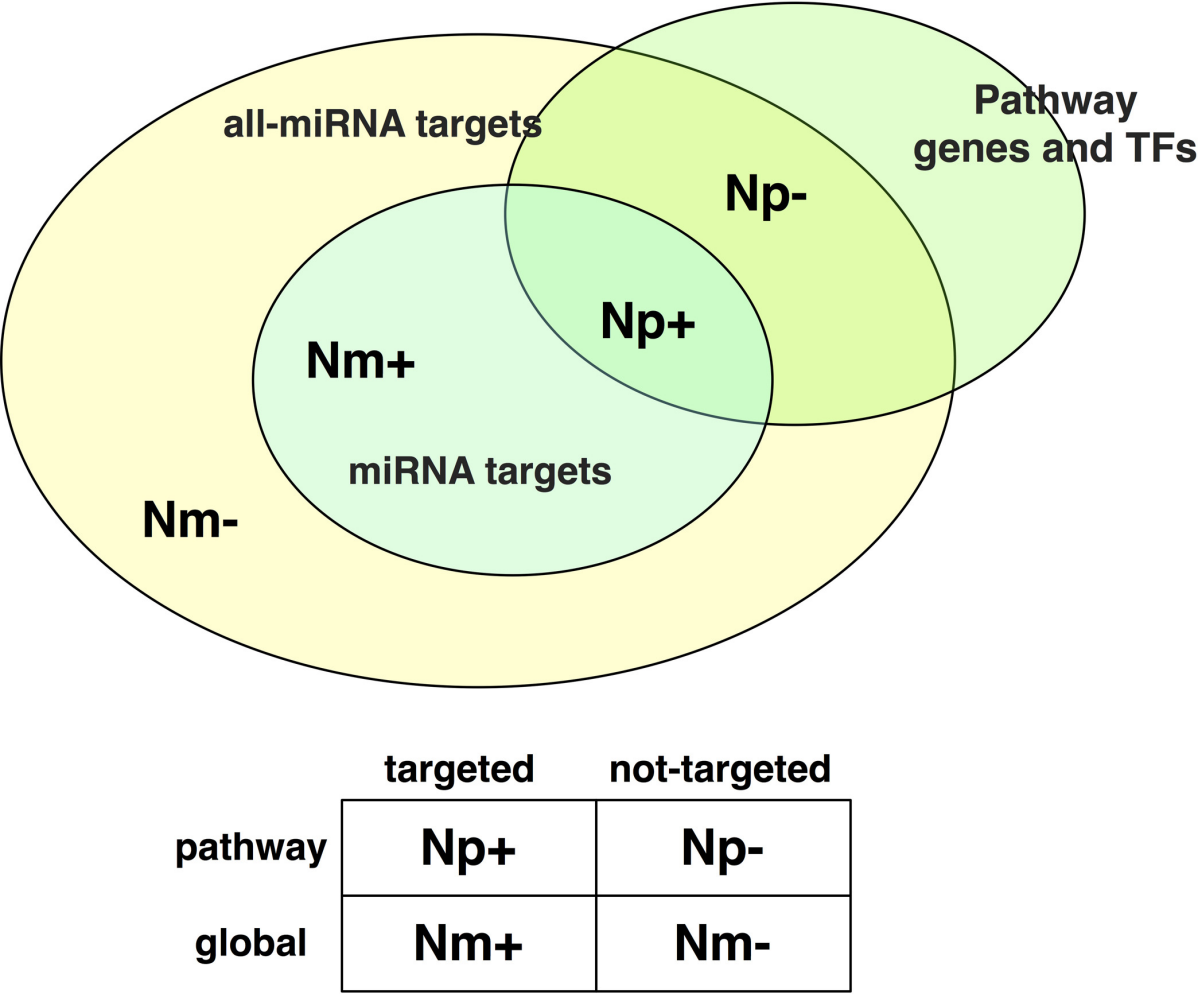
miRNA vs. pathway contingency table. Np+: number of targeted genes in the pathway, Np-: number of non-targeted genes in the pathway, Nm+: number of total targets of the miRNA, Nm-: number of total genes not targeted by the miRNA.

Making use of a combination of php and R scripts, we constructed a 2x2 contingency matrix for each miRNA-pathway pair, as shown in Fig 3. Np+ represents the number of genes in the pathway targeted by the miRNA. It is obtained by intersecting information on miRNA targets with the list of genes and TFs for the pathway. Np- represents the number of non-targeted genes in the pathway, while Nm+ represents the total number of miRNA targets. Finally, Nm- represents the total number of genes not targeted by the miRNA.

To perform a fair analysis, Nm- was computed by merging the list of targets of all considered miRNAs and subtracting the targets of the miRNA under evaluation; and Np- was computed by excluding those genes in the pathway that had never been annotated as a miRNA target in the considered database.

For each miRNA-pathway pair we applied a one-tailed Fisher's exact test to compute the enrichment of the miRNA targets in the selected pathway [36][37]. The one-tailed test for a 2x2 case computes p-values directly using the hypergeometric distribution. It provides exact probabilities and is appropriate for small number statistics [37]. By means of the Fisher's test we were able to test, for each miRNA-pathway pair, the hypothesis of a miRNA having its target genes enriched in a given pathway.

Finally, significance levels computed for each miRNA when paired with each pathway were combined together in order to test the significance of the miRNA enrichment over the joint set of pathways. First, false discovery rate (FDR) was applied to the p-values for each miRNA-pathway pair, in order to correct for multiple-hypothesis testing, thus implementing the correction as previously described in [38]. Second, corrected p-values were combined by applying the modified Lancaster's method proposed in [39]. Lancaster's method is a meta-analysis algorithm, which can be used to combine the results of more than one test bearing upon the same hypothesis. Compared to the Fisher's combined probability test widely used in other enrichment studies to combine p-values [40], Lancaster's method enables the introduction of weighting functions for the combined p-values. Weighting functions allowed for the incorporation of prior biological information in the analysis leading to more meaningful results. In particular, in our study, p-values were weighted according to the relevance class of the related pathway, reported in the S1 Table. Class A pathways where weighted the highest (2), equal to the traditional Fisher's combined probability test [39]. Class B pathways were weighted 1 to account for reduced relevance in the analysis and, finally, class C pathways were weighted 0.5. Finally, the modified Lancaster's method previously proposed in [39] and applied in this study accounts for correlation among the single tests. This is important in our setup since pathways often share common sets of genes. Traditional Fisher's combined probability tests are based on the independence assumption, which does not hold in our setup. Based on the result of the combined p-value test, we were able to assign to each miRNA a single enrichment significance level. Only highly significant enriched miRNAs (p<0.01) were retained in the final reduced panel of miRNAs as candidates for laboratory analysis.

**Fig 4:**
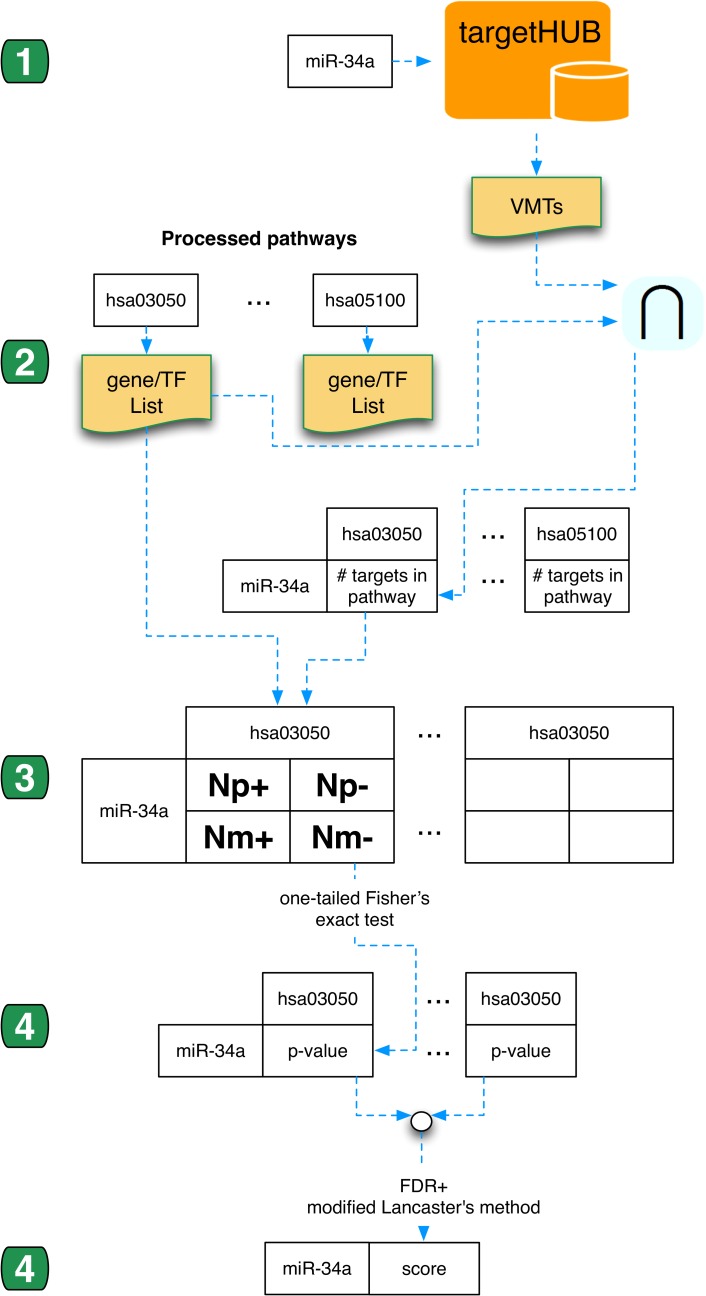
Example of step by step processing of a single miRNA using VMTs. (1) miR-34a, used as an example in the figure, is queried on TargetHUB to obtain its list of targets (VMTs); (2) The list of targets is intersected with the list of genes for each considered pathway to obtain a matrix showing how many targets belong to each pathway; (3) The matrix built during step 2, together with the total gene count for each pathway, is used to construct a set of contingency tables (one for each miRNA/pathway pair) as shown in Fig 3; (4) For each miRNA/pathway pair, the related contingency table is used to perform a one-tailed Fisher’s exact test to compute the enrichment of the miRNA targets in the pathway, obtaining a p-value that measures the significance of the test; and (5) p-values obtained for each miRNA/pathway pair are combined into a single significance score by the application of the modified Lancaster's method proposed in [39].

As an example, Fig 4 reports a sample of the computations performed in the analysis of a single miRNA (miR-34a) using VMTs. The analysis starts from acquisition of the list of miRNA targets and ends with the computation of an enrichment score for the miRNA in the considered set of pathways. The source code for the scripts used to perform this analysis is available in the “4-Enrichment-Analisys” folder of the BOS-miRNA-Enrichment repository on GitHub.

### Final filtering

Finally, the scored list of miRNAs was filtered according to two different criteria. First we selected the miRNAs identified as being expressed in relevant tissue samples. This step is important to focus on the miRNAs specifically active in the target tissue. To perform this operation, we consulted mimiRNA, a database of miRNA expression across different tissues and cell lines [41]. mimiRNA incorporates a sample classification algorithm that groups identical miRNA or mRNA experiments from separate sources. This enables mimiRNA to provide reliable expression profiles and to discover functional relations between miRNAs and mRNAs such as miRNA targets. The mimiRNA database for the selected tissues was downloaded and processed by means of a php script in order to extract a list of miRNAs significantly expressed in lung tissues and in the following cells involved in specific immunity and non-specific inflammation in BOS:

- Lung tissues;
- B lymphocytes;
- Natural killer lymphocytes;
- Monocytes;
- Dendritic cells;
- Granulocytes; and
- A549 (A549 cells derive from a peripheral well-differentiated lung adenocarcinomas, which retain several features of mature deep airway epithelium, and are therefore frequently used in experimental studies as a "model" of human bronchiolo-alveolar epithelium).

The second step was to select intragenic miRNA co-expressed by genes or transcription factors in the selected pathways. To perform this operation, we consulted the miRIAD Intragenic Microrna database [42]. Each gene and transcription factor belonging to the considered pathways was automatically queried on miRIAD to identify if it potentially hosts an intragenic miRNA. Intragenic miRNAs selected in this step are likely to be co-expressed with their host genes [43] and therefore have a good chance of being expressed within biological processes involved in the pathogenesis of BOS.

The full list of 349 miRNAs selected along with the tissue or cell in which they are expressed is reported in the S1 File, while the code for the script used to perform the filtering is available in the “3-Mirna-Processing” folder of the BOS-miRNA-Enrichment repository on GitHub.

### Wet lab experiments

ISH for two candidate miRNAs (*miR-34a* and *miR-*21) was performed on formalin-fixed/paraffin-embedded samples of normal human and rat lungs, lung explants of BOS patients and rat models of acute and chronic lung rejection (outbred CD SPF/VAF), and on mesenchymal cells obtained from bronchoalveolar lavage (BAL) of lung recipients. The specimens were collected from the University of Padua and Foundation IRCSS San Matteo/University of Pavia Pathology Units. The study to develop an animal model of obliterans bronchiolitis was approved by the Research Ethics Committee of University of Padua (Protocol n. 0004959: Approval of informed consent from patients for tissue storage and research use. Date of approval: 27/01/2011. Project n. 23/2014: 244 Development of an animal model of obliterans bronchiolitis. Date of approval: 245 13/10/2014). All procedures used in this study were conformed to the rules and principles of the 2010/63/EU Directive. Lung explants of BOS patients were collected from IRCSS San Matteo/University of Pavia Pathology Units. The study was approved by Pavia Area Ethics Committee of Foundation IRCCS San Matteo (Protocol n. 20140003328: Isolation and characterization of mesenchymal cells obtained from bronchoalveolar lavage of lung recipients. Date of approval: 28/07/2014). Written informed consents of patients have been obtained before explant. Additional information regarding the Research Ethics Committee are provided in S2 File.

miR-34a and miR-21 are double-DIG labeled miRCURY LNA®microRNA Detection Probes and have the sequences 5'-ACAACCAGCTAAGACACTGCCA-3', and 5'-TCAACATCAGTCTGATAAGCTA-3' respectively. U6 and a scramble probe were used respectively as positive and negative controls. All probes were purchased from Exiqon.

ISH was performed according to the manufacturer's protocol provided by Exiqon (Exiqon, Vedbaek, Denmark) [44] with slight modifications described below.

ISH experiments were performed on tissue sections and mesenchymal cells (MCs) isolated from BAL of BOS patients and cultured on Lab-Tek®II Chamber SlidesTM (Thermo Scientific) according to [45]. Normal skin fibroblasts (NHDF-cor; PromoCell GmbH, Heidelberg Germany) were used as control. Six micron thick unstained tissue sections were deparaffinized and digested with Proteinase-K (15 *μ*g/mL) for 10' at 37°C using an Abbott Molecular StatSpin ThermoBrite Hybridizer System (Dako hybridizer). Hybridization with 40nM *miR-21* probe, 1nM U6 probe and 20nM scramble probe was carried out at 50°C for 60 min. For *miR-34a*, a less-abundantly expressed miRNA, 100nM labeled probe was used and hybridization was carried out at 37°C for 20h. Furthermore, to prevent *miR-34a* release and extra-tissutal diffusion during hybridization, an additional miRNA fixation step, using 1-ethyl-3-(3-dimethylaminopropyl) carbodiimide, was added on tissue sections to anchor miRNAs into the protein matrix [46]. Stringent washes were carried out in 5X SSC, 1X SSC and 0.2X SSC at 55°C. The digoxigenins were then recognized by a specific anti-DIG antibody, directly conjugated with the enzyme Alkaline Phosphatase and the cells were stained with NCIP/NBT chromogenic substrate.

Images of miRNA signals were captured by an Olympus BX41 microscope equipped for conventional bright light and epifluorescence microscopy and connected to an image acquisition software (Cell F, Olympus). Consecutive unstained sections were stained with Movat pentachrome stain for connective tissue to highlight tissue morphology and connective tissue components, and immunoreacted with anti-actin HHF35 monoclonal antibody (Dako, Glostrup, DE) on the Dako Omnis autostainer platform to confirm the myofibroblast location of positive ISH signals. All reactions were analyzed under the light microscope and reviewed blindly by two skilled pathologists (PM and FC).

## Results

### Panel of miRNA involved in the pathogenesis of BOS

**Fig 5:**
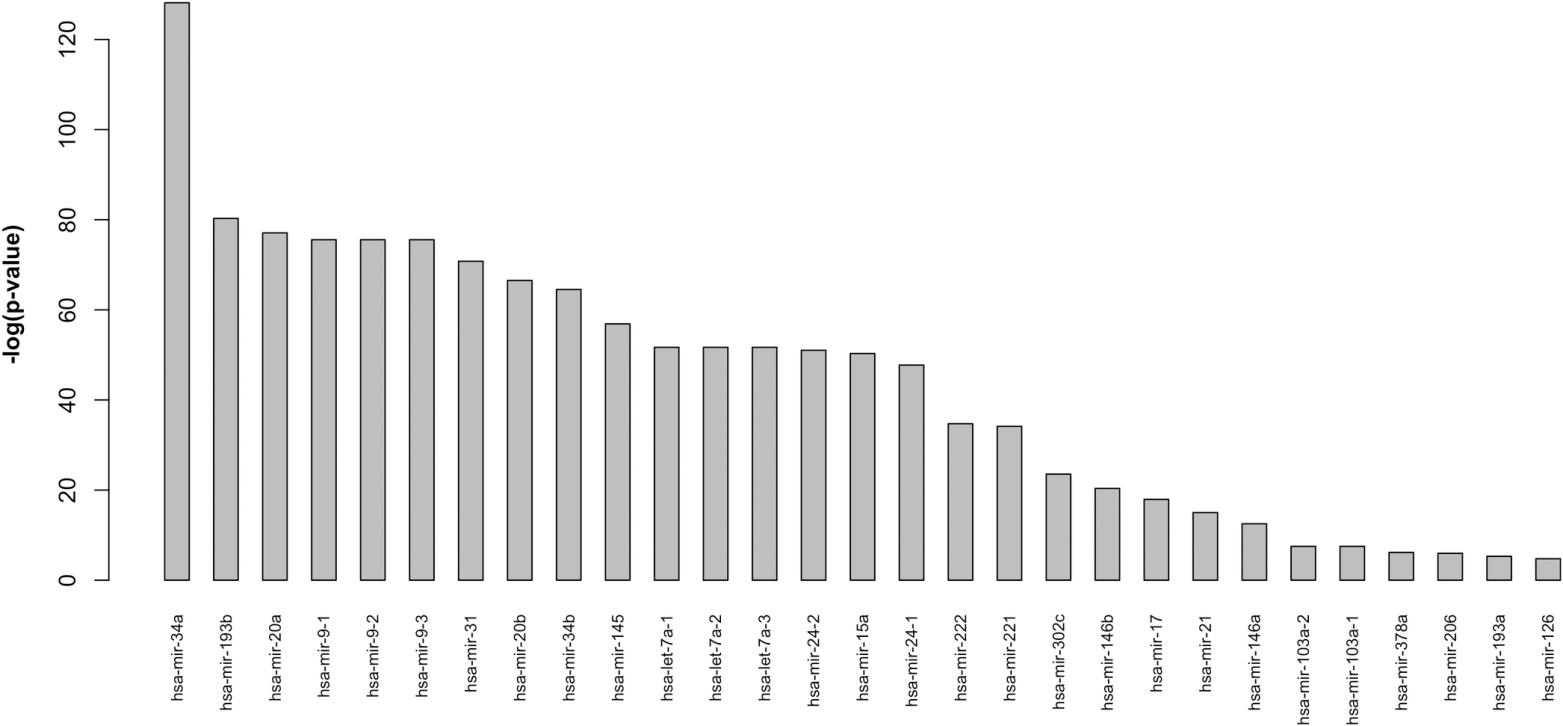
Ranked list of miRNAs most significantly enriched in BOS-related pathways (p<0.01) considering VMTs. Results are plotted using a -log(p-value).

**Fig 6:**
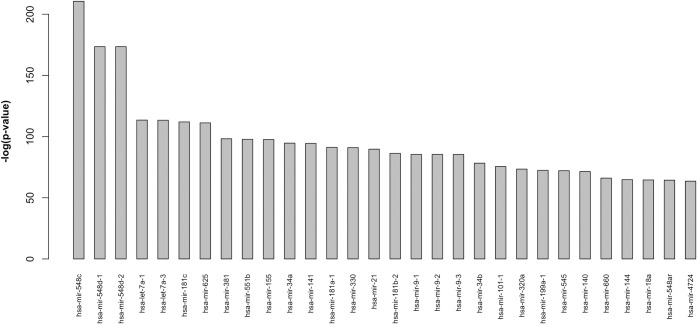
Ranked list of the top 30 miRNAs most significantly enriched in BOS-related pathways (p<0.01) considering CMTs. Results are plotted using a -log(p-value).

Figs 5 and 6 report the main results obtained by the computational pipeline described in Fig 1.

The analysis performed with VMTs (Fig 5) identified a set of 29 miRNAs, out of the 349 miRNAs considered in the initial dataset, whose targets were significantly enriched (p-value<0.01) in the selected pathways. miRNAs were ranked according to the significance of their enrichment in the pathways. This result enabled us to narrow down considerably the set of candidate miRNAs to be investigated in the laboratory. The complete list of scores for the miRNAs considered is presented in the S1 File.

Limiting the analysis to VMTs might have biased the results due to the limited amount of information available in the miRTarBase repository. To enlarge the exploratory scope of our study, we also submitted CMTs to analysis, and we were able to identify another ranked set of 174 miRNAs (Fig 6) whose targets (both computational and validated) were significantly enriched (p-value<0.01) in the set of selected pathways. The list produced in this case was, of course, larger than the one presented in Fig 5, since, unlike VMTs, CMTs are available for all considered miRNAs. This enabled us to enlarge the spectrum of possible interactions between miRNAs and pathways, and to account for miRNAs that might have escaped our attention. Nevertheless, the information obtained in this second analysis must be considered carefully. It is well known that computational methods for miRNA target analysis are not highly accurate and often generate a high number of false positives [47]. The complete list of scores for the considered miRNAs obtained with this second analysis is also available in the S1 File while Fig 6 shows the 30 top ranked miRNAs in this list.

Finally, in order to complete the analysis, we performed a random control experiment by applying the computational pipeline to a random selection of 39 pathways from KEGG.

**Fig 7:**
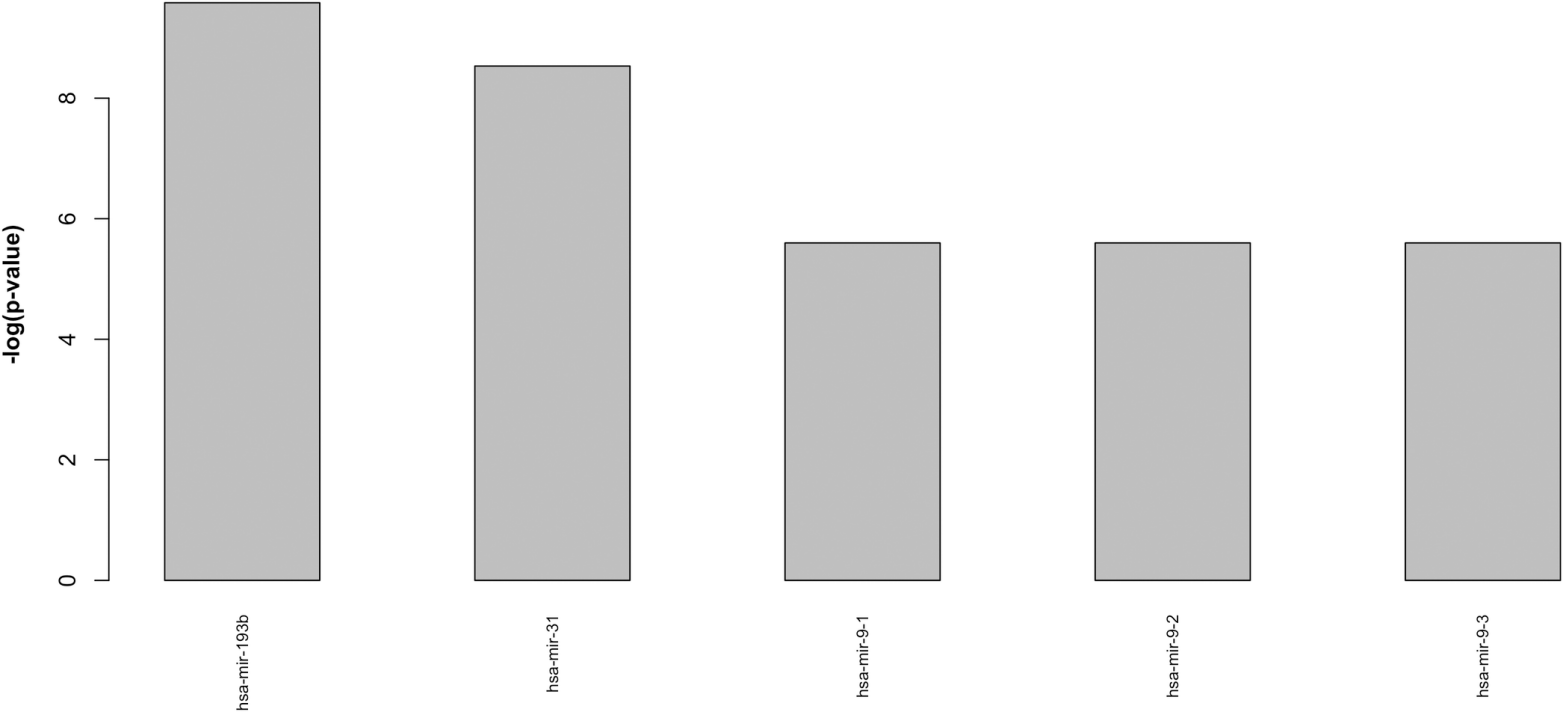
Ranked list of miRNAs highly significantly enriched in a set of 39 randomly selected pathways (p<0.01) considering VMTs. Results are plotted using a -log(p-value).

**Fig 8:**
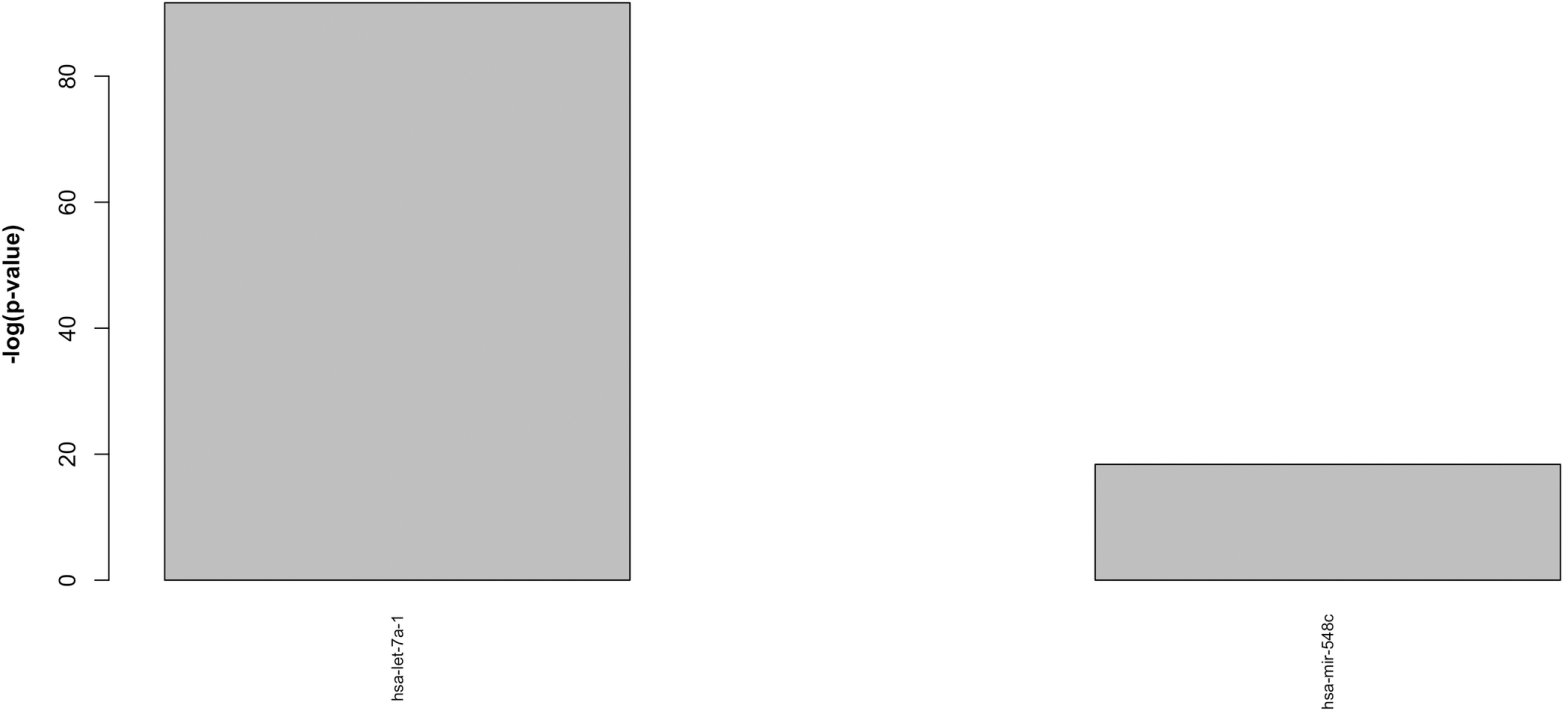
Ranked list of miRNAs highly significantly enriched in a set of 39 randomly selected pathways (p<0.01) considering CMTs. Results are plotted using a -log(p-value).

Interestingly, the number of significantly enriched miRNA selected by the computational flow dropped to only 5 in the analysis performed with VMTs (Fig 7) and to only 2 miRNAs for the analysis performed with CMTs (Fig 8). This provides a good indication that the selected miRNAs depend on the considered set of BOS-related pathways. However, it is interesting to note that in both cases (VMTs and CMTs), the miRNAs identified in the random control were also reported in the list of miRNAs related to BOS. This result was not unexpected. First of all, the reader may note that the score obtained for this random control (-log(p-value)) is higher than the corresponding score assigned when considering BOS related pathways. This means reduced enrichment in the selected set of random pathways. Second, the presence of these miRNAs in the random control is probably an indication that they might be related to more general cell proliferation/differentiation mechanisms relevant in several disease processes. While they can still be found dysregulated in BOS patients (for this reason they have not been removed from the lists reported in Figs 5 and 6) they are less specific for the target disease.

### ISH results

In order to validate the results of the computational analysis, we analyzed specific miRNA expression profiles in a set of lung tissue samples obtained from animals and patients with BOS and in mesenchymal cells from BAL of lung recipients, and compared these with results obtained from normal controls. Information regarding the Research Ethics Committee is provided in the S2 File. The ISH approach was preferred to qRT-PCR, since it not only provides evidence of local dysregulation but also allows morphological correlations and highlights the expression profiles in different cell types (inflammatory cells like macrophages or lymphocytes, normal or reactive epithelial cells, endothelial cells, and, finally, fibroblasts). Specifically, BO lesions involve a small proportion of lung tissue and therefore the quantitative approach could miss significant differences in expression levels occurring in limited, although extremely disease-specific, tissue components.

To choose the first two miRNAs to be validated by wet-lab experiments we firstly focused on those factors present in both lists shown in Figs 5 and 6, but not identified in the random control set of miRNAs. The factors present in both lists were: *let-7a*, *miR-34a*, *miR-21* and *miR-9* family. The *let-7a* and *miR-9* miRNAs were also among those identified in the random control set, and we excluded them as their dysregulation might be related to a more general cell proliferation/differentiation mechanism relevant in several disease processes, but not specifically related to BOS. Moreover, *miR-21* and *miR-34a* had not yet been described in BOS in previous human studies. Given these premises, we selected *miR-34a*, the top-ranked miRNA in Fig 5 (VMTs) and *miR-21*, as candidate miRNAs for ISH analysis.

**Fig 9:**
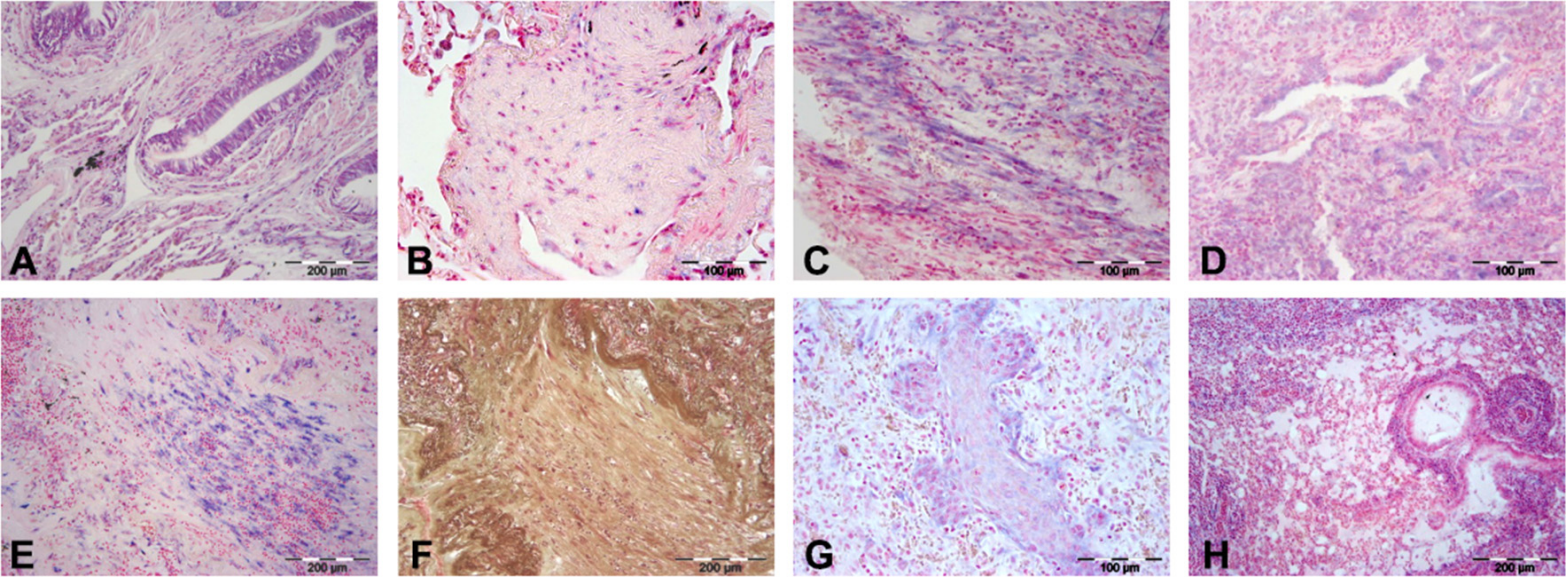
*miR-34a* and *miR-21* expression in human and rat transplanted lungs. Upper panel: *miR-34a* expression in bronchial epithelial cells in normal human lung (A, blue staining), in myofibroblasts in lung explants from a BOS patient (B), in proliferating fibroblasts in rat lungs with chronic rejection (C) and in epithelial cells in remodeled areas in the animal model of acute cellular rejection (D). Lower panel: *miR-21* ISH (E) and Movat pentachrome stains (F) highlighting *miR-21* expression in BO myofibroblasts in lung explants from BOS patients. The airway lumen, which can be recognized by the peripheral elastic fibers, is totally occluded by collagen deposition and myofibroblasts (F), which were strongly positive for *miR-21* (E, blue staining). In rat lungs with chronic rejection, *miR-21* expression was similarly observed in BO myofibroblasts (G), while in acute cellular rejection, *miR-21* expression was localized in epithelia and in interstitial fibroblasts associated with inflammatory infiltrates (H).

Our preliminary results documented the dysregulated expression of *miR-34a* and *miR-21* in human and rat transplanted lungs with BOS (Table 1). Specifically, ISH analysis showed that in normal human and rat lungs, *miR-34a* was diffusely expressed in bronchial and alveolar epithelial cells and in some inflammatory cells, mostly plasma cells (Fig 9A). In lung explants from BOS patients, *miR-34a* was strongly expressed in bronchiolar and reactive alveolar cells and moderate expression was also detectable in proliferating fibroblasts of BO lesions (Fig 9B).

**Table 1:** Profile and expression analysis of *miR-34a* and *miR-21* in human and rat lung samples: normal lung, human chronic rejection (BOS), acute and chronic rat graft rejection.

Human	CTR(1)	ACUTE REJECTION(1)	BOS(5)
*miR-34a*	Bronchial and	//	BO fibroblasts (+++),
	alveolar epithelia (++)	//	Reactive pneumocytes (++),
		//	Bronchial and alveolar epithelia (++)
*miR-21*	Absent	//	BO fibroblasts (+++),
		//	Reactive pneumocytes (++)
Rat	CTR(2)	ACUTE REJECTION(1)	CHRONIC REJECTION(1)
*miR-34a*	Bronchial and	Bronchial and	Reactive pneumocytes (++),
	alveolar epithelia (++)	alveolar epithelia (++),	Fibroblasts (++),
		Inflammatory infiltrates (++)	Bronchial and alveolar epithelia (++),
			Endothelia (++)
*miR-21*	Absent	Reactive pneumocytes (+),	Reactive pneumocytes (++),
		Fibroblasts (++),	Fibroblasts (+++)
		Inflammatory infiltrates (+)	

BO: bronchiolitis obliterans; (+): scarce expression; (++): moderate expression; (+++): strong expression.

Analogously, in the rat orthotopic lung transplantation with chronic bronchiolar rejection, *miR-34a* was strongly expressed in proliferating fibroblasts (Fig 9C), bronchial epithelial cells, endothelia and inflammatory cells, mostly plasma cells, while in the setting of acute cellular rejection, *miR-34a* expression was detectable only in epithelial cells and in inflammatory infiltrates (Fig 9D).

As far as *miR-21* was concerned, human and rat lungs with chronic bronchiolar rejection showed strong expression in the fibroblasts of bronchiolar lesions (Fig 9E and 9G) and in epithelial cells in remodeled areas, while *miR-21* expression was not detectable in either human or animal normal lungs. In the acute rejection animal model, *miR-21* was modestly expressed in epithelia and in interstitial fibroblasts associated with inflammatory infiltrates (Fig 9H). All observations of *miR-34a* and *miR-21* expression were validated with alpha 1-actin and Movat (Fig 9F) connective tissue stains on consecutive sections.

ISH results on mesenchymal cells from BAL confirmed the dysregulation observed in tissue samples. Specifically, *miR-21* was not expressed in normal fibroblasts, while it was modestly expressed in BOS MCs. *miR-34a* expression was evident both in control cells and BOS MCs (data not shown).

## Discussion and Conclusions

In this paper we applied for the first time a complex computational pipeline to perform enrichment analysis of miRNA targets in pathways involved in BOS pathogenesis. The analysis, which started with literature mining to identify a limited set of relevant pathways and integrated information from several public databases, ultimately produced a ranked panel of miRNAs potentially involved in BOS pathogenic process. The analysis considered the full set of miRNAs annotated in miRBase (version 21), and applied a sequence of filtering approaches and statistical analyses to reduce this set and rank the identified miRNAs. The full computational pipeline consulted only publicly available miRNA interaction data, and did not require any expression data to perform its prediction. It was therefore well suited to perform a preliminary screening before conducting actual laboratory experiments.

Two different lists of miRNAs were produced: a first, smaller one, based on VMTs; and a second, larger one, based on CMTs.

We proceeded to validate the analysis of the ranked lists of miRNAs by means of wet lab experiments (ISH) on two highly-ranked miRNAs that appeared in both lists: *miR-34a* and *miR-21*. As stated above, they were chosen among those not yet described in the most recent literature on BOS; and because of their presence in both lists (validated and computational factors).

**Fig 10:**
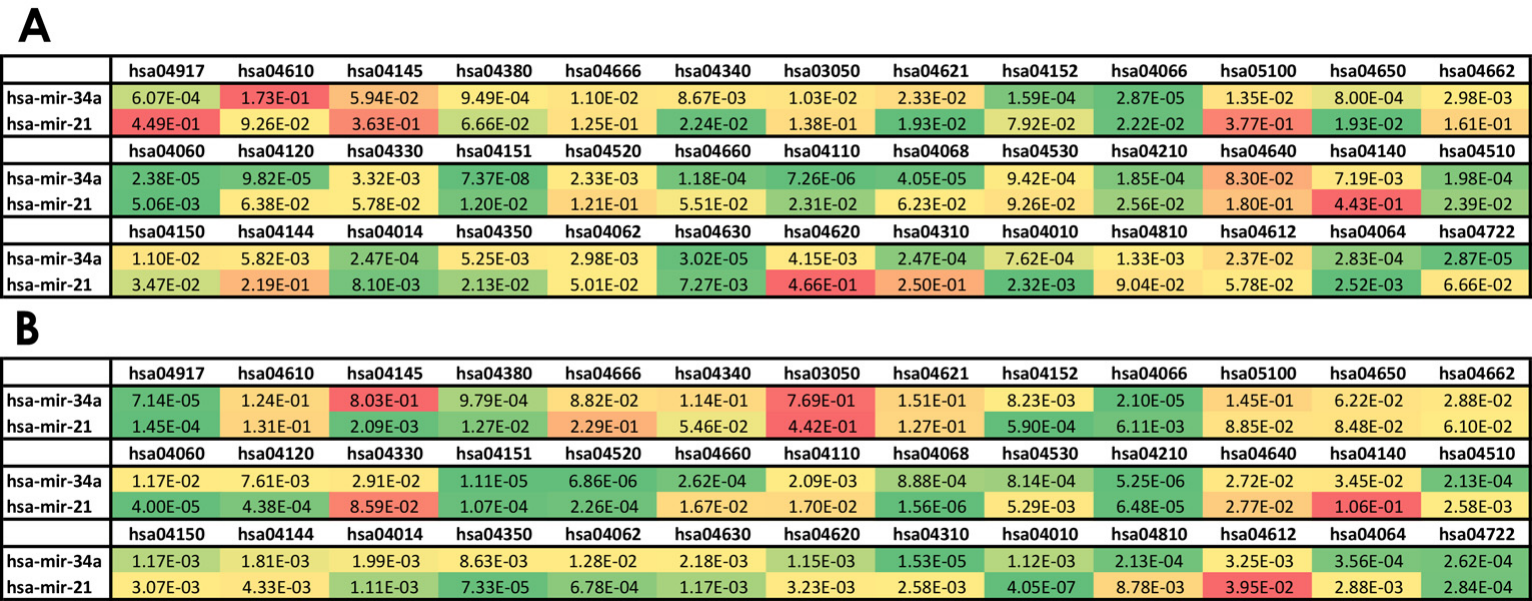
P-values obtained from the miRNA targets enrichment analysis for *miR-34a* and *miR-21* paired with all considered pathways. The color scale indicates the significance of the enrichment (green = significant enrichment). A: analysis with VMTs; B: analysis with CMTs.

Fig 10 shows the p-values obtained from the enrichment analysis of these two miRNAs against all considered pathways. Subfigure A reports data computed with VMTs while subfigure B reports data computed with CMTs. These p-values have been used to compute the scores used to rank the miRNAs in Figs 5 and 6.

The first factor chosen for the validation phase was *miR-34a*, which ranked first in the VMTs list (Fig 5) and eleventh in the CMTs list (Fig 6). It is an intergenic factor that has been reported as a tumor suppressing gene, being consistently downregulated (mainly by promoter hypermethylation) in a variety of cancer cells (breast, prostate, bladder, pancreas, kidney and colon carcinoma, glioblastoma, myeloma, melanoma, and neuroblastoma) [48][49][50]. We were able to document diffuse expression of *miR-34a* in bronchial and alveolar epithelial cells, as well as in endothelial and inflammatory cells of normal adult lungs.

In lung samples obtained from BOS patients, *miR-34a* was clearly expressed in normal bronchiolar, alveolar epithelia and reactive pneumocytes, but it was also expressed in proliferating fibroblasts, suggesting that its dysregulated expression might play a role in the fibrogenic process of BOS.

Published evidence of a profibrogenic role of *miR-34a* and of its expression by proliferating fibroblasts is however very limited. It has been reported as being upregulated in macrophages in the bleomycin model of pulmonary fibrosis [51] and it has been shown to play a critical role in the progression of cardiac tissue fibrosis, mainly by targeting the *TGF-beta*/*SMAD* signal transduction pathway [52].

Recently, *miR-34a* and *miR-34c* have been shown able to down regulate peroxisome proliferator-activated receptor (*PPAR*) in hepatic stellate cells thus inducing their activation, a hallmark of liver fibrosis [53]. *PPAR* has, on the other hand, been recognized as a relevant anti-fibrotic mediator (mainly by inducing the inhibition of *TGF-beta*/*SMAD* signal transduction pathway), thus its down regulation might be important in driving fibroblast proliferation and extracellular matrix deposition, as recently suggested in an investigation of systemic sclerosis-associated skin and lung fibrosis [54]. Further data are required to clarify the molecular mechanisms underlying the pro-fibrotic role of *miR-34a* in BOS that we suggest in this study.

The second factor, *miR-21* ranked twenty-second in the VMTs list (Fig 5) and fifteenth in the CMTs list (Fig 6).

Interestingly, our ISH results revealed a net overexpression of *miR-21* in BO lesions: it was primarily expressed in fibroblasts and in activated epithelial cells in all human BOS cases and in rat grafts, while it was completely absent in normal human and rat lungs, thus clearly demonstrating upregulation in BO. Literature data in support of role of *miR-21* in fibrogenesis are more consistent. It was found to be significantly upregulated in the lung tissue of animal models of bleomycin-induced pulmonary fibrosis [55]; and upregulated 2.7-fold by RT PCR in a mouse model of orthotopic tracheal transplantation [4]. In addition, *miR-21* upregulation has been demonstrated in fibroblast foci as well as in the serum of patients with idiopathic pulmonary fibrosis, with a significant correlation between serum levels and the degree of lung function impairment [56]. Besides its involvement in fibro-proliferative processes in several organs [54][56][57][58][59][60][61], *miR-21* has been shown to play an important role in several other physiological and pathological processes [62]. Its up-regulation has been observed in solid tumors (breast, colon, lung, pancreas, prostate and stomach, ovarian, cervical, head and neck carcinomas), in leukemias and in a variety of other human proliferative disorders, implying a function in regulating cell growth [62].

The mature *miR-21* sequence is strongly conserved throughout evolution and is encoded by a single intergenic gene located on the plus strand of chromosome 17q23.2, where it overlaps with the protein-coding gene *VMP1*. Its transcription is under the control of *AP-1*, *SRF*, *p53*, Signal Transducer and Activator of Transcription 3 (*STAT3*) and many other TFs, as well as of epigenetic mechanisms [62]. It is also controlled at the post-transcriptional level by: *TGF-beta* and *BMP* via *SMAD1/5* and *SMAD2/3*. Since the latter signaling pathway is important in epithelial-mesenchymal transition (EMT), it is reasonable to think that *miR-21* plays a role in this process [62]. Moreover, hypoxia, which is a well-recognized stimulus for EMT [63], is a potent *miR-21* inducer. Indeed, an *HIF-1alpha* binding site is present in the pri-*miR-21* promoter [64]. *miR-21* targets several factors responsible for cell growth and invasion suppression, cell cycle arrest, MMP and other protease inhibition, control of angiogenesis, cellular branching and migration, with a variety of regulatory feedback loops [62]. Their down-regulation has been reported in several cancers in which *miR-21* is overexpressed [62]. Finally, *miR-21* dysregulation has been demonstrated in bio-fluids [65][66] and anti-*miR-*21 has been used as targeted therapy in cancer, obtaining promising results both in vitro and in vivo [67,68].

On the basis of our novel finding and of previous evidence of the role of *miR-21* in homeostasis and disease, its contribution to BOS pathogenesis can be inferred to be at the level of myofibroblast migration and proliferation in the airway lumen, while a possible additional regulatory activity in the mechanism of epithelial-mesenchymal transition cannot be excluded.

Overall, this is the first study to systematically analyze and score a large number of miRNAs implicated in BOS. We were able to process all the miRNAs currently annotated in miRBase (version 21) and, by means of a sequence of computational filtering methods, we were able to filter them and then rank them according to their enrichment into the selected pathways.

In addition to *miR-34a* and *miR-21*, other miRNAs have recently been described as being dysregulated in BOS. Two recent papers reported an integrated analysis of miRNA expression in the mouse models of orthotopic tracheal transplantation [4] [5]. The following miRNAs, also present in the VTMs list of Fig 5, were found by RT-PCR to be dysregulated in mouse lung tissue: *miR-21*, *miR-146*, *miR-20*, *miR-302*, *miR-19*, *miR-98*, *let-7a*, *miR-15a*. In comparison with lung recipients without BOS, clear dysregulation of *miR-34a*, *miR-193b*, *miR-9* and *miR-15a*, likewise present in the VTM list in Fig 5, was also detected in peripheral mononuclear cells obtained from BOS patients in a RT-PCR evaluation of miRNA expression by Xu at al. [3]. Even if the above results were obtained on the one hand in animals by RT-PCR without a comprehensive analysis of cell type-restricted expression; and on the other in humans but on a specific peripheral inflammatory cell subset, without further confirmation on lung tissue, they might support the validation of our pipeline. Further analyses are, however, necessary to confirm the role of these factors in the pathogenesis of BOS.

The main limitation of our study is that only two factors identified by our pipeline underwent validation. However, since our wet-lab experiments were intended only to confirm the results obtained with the computational approach, more extensive tissue analysis was beyond the scope of the study. In addition, we chose 2 miRNAs which had never been associated with BO in previous human studies. For these reasons, while we did demonstrate *miR-34a* and *miR-21* dysregulation in fibroblasts obliterating the bronchiolar lumen, we cannot provide mechanistic insights into their role in BOS pathogenesis, and these should be addressed in specifically designed studies.

**Fig 11:**
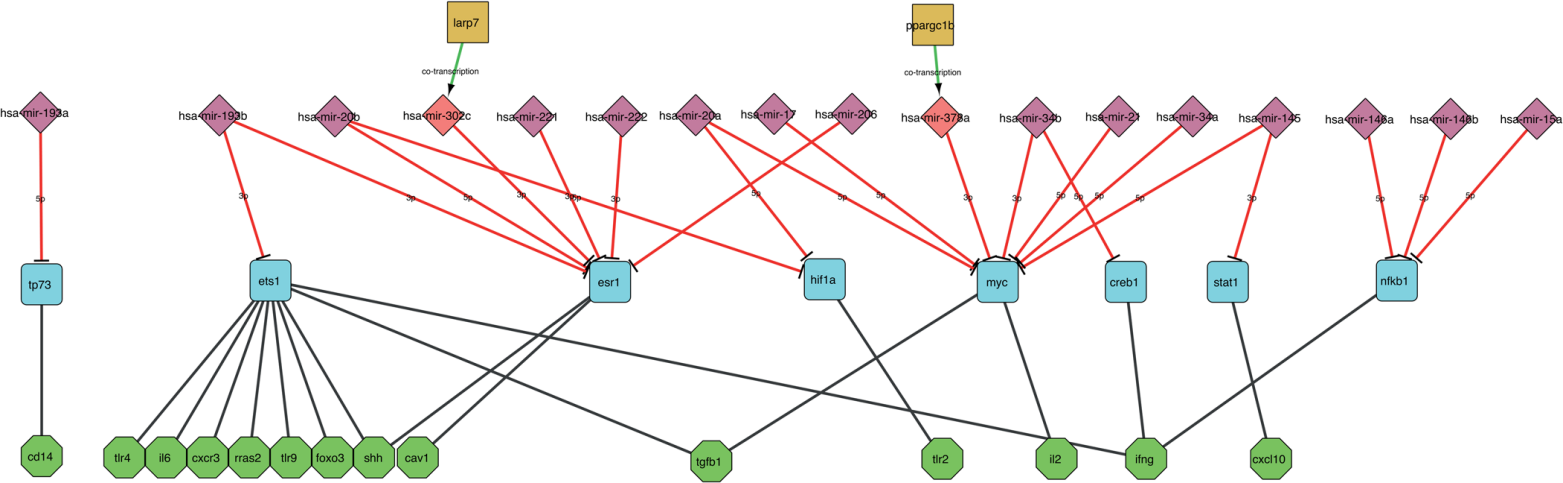
Gene regulatory network showing interaction between miRNAs identified in the VMTs list and relevant genes implicated in BOS as identified from the literature (see the S1 Table). The network is hierarchically organized into four levels. Brown squares: miRNA host genes for intragenic miRNAs; red and purple rhombi: intragenic and intergenic miRNAs respectively; cyan squares TFs; green hexagons: target genes.

Nevertheless, the data depicted in Fig 11 are a preliminary step in this direction. This shows a regulatory network depicting the interactions between miRNAs identified in the VMTs list in Fig 5 and all genes identified during the initial literature search as being involved in BOS (see S1 Table). The network was constructed using the CyTransfinder Cytoscape plugin [69]. Interestingly, 17 out of the 29 identified miRNAs were involved in the regulation of 14 out of the 27 identified genes. Even more interestingly, none of the identified relations involves a direct interaction between a miRNA and a target gene, but all are mediated through one transcription factor introduced while processing the considered pathways. All miRNAs and genes not included in this network involve longer chains of regulation that we could not easily reconstruct through the CyTransfinder tool and require further investigation.

## Supporting Information

S1 File This file contains the final significance scores (VMTs and CMTs) computed for the analyzed miRNAs including the tissue or cell in which they are expressed. (XLSX)

S2 File This file contains research ethics committee information regarding the experiments described in the paper. (PDF)

S1 Table List of analyzed pathways. For each pathway the table reports: the unique KEGG Identifier, the pathway name, the assigned class based on its relevance, the relevant genes and the related references that identify the pathway as relevant for BOS.
(PDF)
