# Supplementary material for: Identification of miRNAs Potentially Involved in Bronchiolitis Obliterans Syndrome: A Computational Study
Source: PLoS One. 2016 Aug 26;11(8):e0161771. doi: 10.1371/journal.pone.0161771 (PMC5001701; doi:10.1371/journal.pone.0161771)
Supplement: S2 File — (PDF) [file pone.0161771.s002.pdf]

S3 File: Research ethics committee information

The study was performed on formalin-fixed/paraffin-embedded samples of normal human and rat lungs, lung explants of BOS patients and rat models of acute and chronic lung rejection , and on mesenchymal cells obtained from bronchoalveolar lavage (BAL) of lung recipients.

The study was approved by the Research Ethics Committee of University of Padua (Protocol n. 0004959: Approval of informed consent from patients for tissue storage and research use. Date of approval: 27/01/2011. Project n. 23/2014: 244 Development of an animal model of obliterans bronchiolitis. Date of approval: 245 13/10/2014). All procedures used in this study were conformed to the rules and principles of the 2010/63/EU Directive. Rat samples were collected from the University of Padua. Animal model was constituted by outbred Sprague-Dawley rats female, provided from Charles River (Germany), weighing between 250 and 350 g and 8-10 weeks old. Animals were single-housed with food and water freely available, maintained on 12-h light/dark cycle with a constant temperature (22 ± 2 °C) and humidity (50 ± 5%) conditions, in pathogen-free ambient. All experiments were carried out in manner to minimize suffering; clinical sings were recorder 3 times for day using the dedicated evaluation system (score sheet system [1-3]). This score system was strictly applied to precociously recognize and avoid any sufferance. In case of animal sufferance, rats were euthanized before stated end-point, causes of this event were: primary graft dysfunction, hypertensive pneumothorax, hemothorax, massive hemoptysis, pulmonary edema. For surgery, rats were anesthetized using 100 mg/kg ketamine and 4 mg/kg xylazine intraperitoneally. All recipient rats were additionally pretreated with buprenorphine (0.005 mg/kg) at the induction time and three times a day in the following 7 days to obtain the best possible analgesia. After surgery, rats were single-housed and treated with gentamicin 10mg/kg/day at time of surgery and in the following week. Animals were handled at least thrice a day for 2 min by the dedicated experimenters to the animal care to administer subcutaneous therapies. Rats were euthanized using intraperitoneal general anesthesia (ketamine 50 mg/kg and xylazine 4 mg/kg) intubated with 16G angio-catheter and ventilated on a FiO2 of 0.5. A median sternotomy was performed followed by subsequent intracardiac injection of Tanax® (Enbutramide/Mebezonio iodide/tetracaine) 0.5ml. Heart-lung block was removed from chest cavity and analyzed.

Lung explants of BOS patients were collected from IRCSS San Matteo/University of Pavia Pathology Units. The study was approved by Pavia Area Ethics Committee of Foundation IRCCS San Matteo (Protocol n. 20140003328: Isolation and characterization of mesenchymal cells obtained from bronchoalveolar lavage of lung recipients. Date of approval: 28/07/2014). Written informed consents of patients have been obtained before explant.

References

[1] Morton DB, Griffiths PHM Guidelines on the recognition of pain, distress and discomfort in experimental animals and an hypothesis for assessment. Veterinary Record. 1985, 116, 431–436.

[2] Morton D, A systematic approach for establishing humane endpoints. ILAR Journal 2000 V41(2).

[3] Morton D, Humane Endpoints in Animal Experiments for Biomedical Research: ethical, legal and practical aspects. Proceedings of the International Conference, 22-25 November 1998, Zeist, The Netherlands. Laboratory Animals Ltd, by Royal Society of Medicine Press Limited, London, England 5-12.
