# Supplementary material for: Identification of miRNAs Potentially Involved in Bronchiolitis Obliterans Syndrome: A Computational Study
Source: PLoS One. 2016 Aug 26;11(8):e0161771. doi: 10.1371/journal.pone.0161771 (PMC5001701; doi:10.1371/journal.pone.0161771)
Supplement: S1 Table — For each pathway the table reports: the unique KEGG Identifier, the pathway name, the assigned class based on its relevance, the relevant genes and the related references that identify the pathway as relevant for BOS. (PDF) [file pone.0161771.s003.pdf]

S1 TABLE: For each pathway the table reports: the unique KEGG Identifier, the pathway name, the assigned class based on its relevance, the relevant genes and the related references that identify the pathway as relevant for BOS.

KEGG Id	Name	Class	Associated gene (described in BOS)	References
hsa04010	MAPK signaling pathway	A	TGF-beta, CD14	[1–15]
hsa04014	Ras signaling pathway	A	RAS	[16]
hsa04062	Chemokine signaling pathway	A	CXCL10, CXCL9, CXCR3	[1, 7, 9, 17–21]
hsa04064	NF-kappa B signaling pathway	A	CD14	[11–13, 22, 23]
hsa04066	HIF-1 signaling pathway	A	IL6, IFNG	[1, 15, 24, 25]
hsa04068	FoxO signaling pathway	A	TGF-beta, IL6	[1–10, 15]
hsa04151	PI3K-Akt signaling pathway	A	IL6, TLR2-4-9, IL2	[1, 13, 15, 22, 26]
hsa04152	AMPK signaling pathway	A	FOXO3	[27, 28]
hsa04210	Apoptosis	B	CSF2RB, CSF2RA	[1]
hsa04310	Wnt signaling pathway	A	MMP7	[1, 15, 20, 29]
hsa04350	TGF-beta signaling pathway	A	IFNG, TGF-Beta	[1–10, 15, 30–33]
hsa04722	Neurotrophin signaling pathway	A	NT4/5, TrkB	[34]
hsa04145	Phagosome	C	MBL, CD14, TLR2-4-9, SFTPD, SFTPA2	[1, 11–15, 22, 23, 38–41]
hsa03050	Proteasome	C	IFNG	[15]
hsa04060	Cytokine-cytokine receptor interaction	A	IFNG, TGF-beta, IL6,CXCL10, CXCL9, CXCR3, IL17A, IL23A, IL2, CSF2RA	[1–10, 15, 21, 22, 37]
hsa04140	Regulation of autophagy	A	IFNG	[1, 15, 30–33]
hsa04380	Osteoclast differentiation	C	IFNG, TGF-beta	[1–10, 30–33]
hsa04612	Antigen processing and presentation	A	IFNG, KIR	[1, 15, 30–33, 42]
hsa04630	Jak-STAT signaling pathway	A	IFNG, IL23A, IL6, IL2, CSF2RB, CSF2RA	[1, 15, 22, 26, 30–33]
hsa04650	Natural killer cell mediated cytotoxicity	A	IFNG, KIR	[1, 15, 30–33, 42]
hsa04660	T cell receptor signaling pathway	A	IFNG, IL2	[1, 15, 26, 30–33]
hsa04640	Hematopoietic cell lineage	C	CD14	[11, 12, 15, 22, 23]
hsa04620	Toll-like receptor signaling pathway	A	IL6, CXCL10, CXCL9, CD14, TLR 2-4-9	[1, 11, 12, 14, 15, 21–23]
hsa04810	Regulation of actin cytoskeleton	A	CD14	[11, 12, 15, 22, 23]
hsa04610	Complement and coagulation cascades	C	MBL	[15, 38–40]
hsa04110	Cell cycle	C	TGF-beta	[1–10, 15]
hsa04144	Endocytosis	C	Cav1, TGF-beta	[1–10, 15, 43, 44]
hsa04510	Focal adhesion	A	Cav1	[1, 43, 44]
hsa04621	NOD-like receptor signaling pathway	A	IL6	[1, 15, 22]
hsa04662	B cell receptor signaling pathway	A	Anti HLA Ab	[1, 45–48]
hsa04340	Hedgehog signaling pathway	B	SHH	[1, 49]
hsa05100	Bacterial invasion of epithelial cells	C	Cav1	[1, 43, 44]
hsa04917	Prolactin signaling pathway*	B	FOXO3	[35–37]
hsa04530	Tight junction	B		[1, 50]
hsa04666	Fc gamma R-mediated phagocytosis	C		[1]

*Prolactin pathway has been included since prolactin (PRL) is involved in a wide range of biological functions including immunomodulation and activates many signaling cascades like MAPK and PI3K pathways, which are, instead, closely related to BOS pathogenesis.

KEGG Id	Name	Class	TF count	Associated gene (described in BOS)	References
hsa04120	Ubiquitin mediated proteolysis	C	32		[1]
hsa04520	Adherens junction	B	37		[1, 50]
hsa04330	Notch signaling pathway	A	24		[1]
hsa04150	mTOR signaling pathway	A	44		[1, 51]

References

1. Xu Z, Nayak D, Yang W, Baskaran G, Ramachandran S, Sarma N, et al. Dysregulated MicroRNA Expression and Chronic Lung Allograft Rejection in Recipients With Antibodies to Donor HLA. Am J Transplant. 2015 Jul;15(7):1933–47.
2. Alho HS, Maasilta PK, Vainikka T, Salminen US. Platelet-derived growth factor, transforming growth factor-beta, and connective tissue growth factor in a porcine bronchial model of obliterative bronchiolitis. Exp Lung Res. 2007 Aug;33(6):303–20.
3. Ramirez AM, Shen Z, Ritzenthaler JD, Roman J. Myofibroblast transdifferentiation in obliterative bronchiolitis: tgf-beta signaling through smad3-dependent and -independent pathways. Am J Transplant. 2006 Sep;6(9):2080–8.
4. Ramirez AM, Takagawa S, Sekosan M, Jaffe HA, Varga J, Roman J. Smad3 deficiency ameliorates experimental obliterative bronchiolitis in a heterotopic tracheal transplantation model. Am J Pathol. 2004 Oct;165(4):1223–32.
5. Aris RM, Walsh S, Chalermskulrat W, Hathwar V, Neuringer IP. Growth factor upregulation during obliterative bronchiolitis in the mouse model. Am J Respir Crit Care Med. 2002 Aug;166(3):417–22.
6. Liu M, Suga M, Maclean AA, St George JA, Souza DW, Keshavjee S. Soluble transforming growth factor-beta type III receptor gene transfection inhibits fibrous airway obliteration in a rat model of Bronchiolitis obliterans. Am J Respir Crit Care Med. 2002 Feb;165(3):419–23.
7. Elssner A, Jaumann F, Dobmann S, Behr J, Schwaiblmair M, Reichenspurner H, et al. Elevated levels of interleukin-8 and transforming growth factor-beta in bronchoalveolar lavage fluid from patients with bronchiolitis obliterans syndrome: proinflammatory role of bronchial epithelial cells. Munich Lung Transplant Group. Transplantation. 2000 Jul;70(2):362–7.
8. El-Gamel A, Sim E, Hasleton P, Hutchinson J, Yonan N, Egan J, et al. Transforming growth factor beta (TGF-beta) and obliterative bronchiolitis following pulmonary transplantation. J Heart Lung Transplant. 1999 Sep;18(9):828–37.
9. Bergmann M, Tiroke A, Schäfer H, Barth J, Haverich A. Gene expression of profibrotic mediators in bronchiolitis obliterans syndrome after lung transplantation. Scand Cardiovasc J. 1998;32(2):97–103.
10. Magnan A, Mege JL, Escallier JC, Brisse J, Capo C, Reynaud M, et al. Balance between alveolar macrophage IL-6 and TGF-beta in lung-transplant recipients. Marseille and Montréal Lung Transplantation Group. Am J Respir Crit Care Med. 1996 Apr;153(4 Pt 1):1431–6.
11. Palmer SM, Klimecki W, Yu L, Reinsmoen NL, Snyder LD, Ganous TM, et al. Genetic regulation of rejection and survival following human lung transplantation by the innate immune receptor CD14. Am J Transplant. 2007 Mar;7(3):693–9.
12. Ward C, Walters EH, Zheng L, Whitford H, Williams TJ, Snell GI. Increased soluble CD14 in bronchoalveolar lavage fluid of stable lung transplant recipients. Eur Respir J. 2002 Mar;19(3):472–8.
13. Todd JL, Wang X, Sugimoto S, Kennedy VE, Zhang HL, Pavlisko EN, et al. Hyaluronan contributes to bronchiolitis obliterans syndrome and stimulates lung allograft rejection through activation of innate immunity. Am J Respir Crit Care Med. 2014 Mar;189(5):556–66.
14. Stober VP, Szczesniak C, Childress Q, Heise RL, Bortner C, Hollingsworth JW, et al. Bronchial epithelial injury in the context of alloimmunity promotes lymphocytic bronchiolitis through hyaluronan expression. Am J Physiol Lung Cell Mol Physiol. 2014 Jun;306(11):L1045–55.

15. Kastelijn EA, van Moorsel CHM, Ruven HJT, Lammers JWJ, Grutters JC. Genetic polymorphisms and bronchiolitis obliterans syndrome after lung transplantation: promising results and recommendations for the future. Transplantation. 2012 Jan;93(2):127–35.
16. Watanabe-Takano H, Takano K, Hatano M, Tokuhisa T, Endo T. DA-Raf-Mediated Suppression of the Ras-ERK Pathway Is Essential for TGF-β1-Induced Epithelial-Mesenchymal Transition in Alveolar Epithelial Type 2 Cells. PLoS One. 2015;10(5):e0127888.
17. Borthwick LA, Corris PA, Mahida R, Walker A, Gardner A, Suwara M, et al. TNFα from classically activated macrophages accentuates epithelial to mesenchymal transition in obliterative bronchiolitis. Am J Transplant. 2013 Mar;13(3):621–33.
18. Meloni F, Solari N, Miserere S, Morosini M, Cascina A, Klersy C, et al. Chemokine redundancy in BOS pathogenesis. A possible role also for the CC chemokines: MIP3-beta, MIP3-alpha, MDC and their specific receptors. Transpl Immunol. 2008 Jan;18(3):275–80.
19. Meloni F, Cascina A, Paschetto E, Marone Bianco A, Morosini M, Pellegrini C, et al. Monocyte chemoattractant protein-1 levels in bronchoalveolar lavage fluid of lung-transplanted patients treated with tacrolimus as rescue treatment for refractory acute rejection. Transplant Proc. 2003 Jun;35(4):1523–6.
20. Kastelijn EA, van Moorsel CH, Ruven HJ, Karthaus V, Kwakkel-van Erp JM, van de Graaf EA, et al. Genetic polymorphisms in MMP7 and reduced serum levels associate with the development of bronchiolitis obliterans syndrome after lung transplantation. J Heart Lung Transplant. 2010 Jun;29(6):680–6.
21. Jiang D, Liang J, Guo R, Xie T, Kelly FL, Martinu T, et al. Long-term exposure of chemokine CXCL10 causes bronchiolitis-like inflammation. Am J Respir Cell Mol Biol. 2012 May;46(5):592–8.
22. Belperio JA, DiGiovine B, Keane MP, Burdick MD, Ying Xue Y, Ross DJ, et al. Interleukin-1 receptor antagonist as a biomarker for bronchiolitis obliterans syndrome in lung transplant recipients. Transplantation. 2002 Feb;73(4):591–9.
23. Ross DJ, Cole AM, Yoshioka D, Park AK, Belperio JA, Laks H, et al. Increased bronchoalveolar lavage human beta-defensin type 2 in bronchiolitis obliterans syndrome after lung transplantation. Transplantation. 2004 Oct;78(8):1222–4.
24. Tiriveedhi V, Gelman AE, Mohanakumar T. HIF-1α signaling by airway epithelial cell K-α1-tubulin: role in fibrosis and chronic rejection of human lung allografts. Cell Immunol. 2012;273(1):59–66.
25. Hodge G, Hodge S, Chambers D, Reynolds PN, Holmes M. Bronchiolitis obliterans syndrome is associated with absence of suppression of peripheral blood Th1 proinflammatory cytokines. Transplantation. 2009 Jul;88(2):211–8.
26. Vanaudenaerde BM, De Vleeschauwer SI, Vos R, Meyts I, Bullens DM, Reynders V, et al. The role of the IL23/IL17 axis in bronchiolitis obliterans syndrome after lung transplantation. Am J Transplant. 2008 Sep;8(9):1911–20.
27. Nho RS, Hergert P, Kahm J, Jessurun J, Henke C. Pathological alteration of FoxO3a activity promotes idiopathic pulmonary fibrosis fibroblast proliferation on type i collagen matrix. Am J Pathol. 2011 Nov;179(5):2420–30.
28. Mihaylova MM, Shaw RJ. The AMPK signalling pathway coordinates cell growth, autophagy and metabolism. Nat Cell Biol. 2011 Sep;13(9):1016–23.
29. Kuo E, Bharat A, Shih J, Street T, Norris J, Liu W, et al. Role of airway epithelial injury in murine orthotopic tracheal allograft rejection. Ann Thorac Surg. 2006 Oct;82(4):1226–33.
30. Lu KC, Jaramillo A, Lecha RL, Schuessler RB, Aloush A, Trulock EP, et al. Interleukin-6 and interferon-gamma gene polymorphisms in the development of bronchiolitis obliterans syndrome after lung transplantation. Transplantation. 2002 Nov;74(9):1297–302.
31. Neujahr DC, Perez SD, Mohammed A, Ulukpo O, Lawrence EC, Fernandez F, et al. Cumulative exposure to gamma interferon-dependent chemokines CXCL9 and CXCL10 correlates with worse outcome after lung transplant. Am J Transplant. 2012 Feb;12(2):438–46.
32. Saini D, Weber J, Ramachandran S, Phelan D, Tiriveedhi V, Liu M, et al. Alloimmunity-induced autoimmunity as a potential mechanism in the pathogenesis of chronic rejection of human lung allografts. J Heart Lung Transplant. 2011 Jun;30(6):624–31.
33. Bharat A, Kuo E, Steward N, Aloush A, Hachem R, Trulock EP, et al. Immunological link between primary graft dysfunction and chronic lung allograft rejection. Ann Thorac Surg. 2008 Jul;86(1):189–95; discussion 196–7.

34. Avcuoglu S, Wygrecka M, Marsh LM, Günther A, Seeger W, Weissmann N, et al. Neurotrophic tyrosine kinase receptor B/neurotrophin 4 signaling axis is perturbed in clinical and experimental pulmonary fibrosis. Am J Respir Cell Mol Biol. 2011 Oct;45(4):768–80.

35. Radhakrishnan A, Raju R, Tuladhar N, Subbannayya T, Thomas JK, Goel R, et al. A pathway map of prolactin signaling. J Cell Commun Signal. 2012 Aug;6(3):169–73.

36. Halperin J, Devi YS, Devi SY, Elizur S, Stocco C, Shehu A, et al. Prolactin signaling through the short form of its receptor represses forkhead transcription factor FOXO3 and its target gene galt causing a severe ovarian defect. Mol Endocrinol. 2008 Feb;22(2):513–22.

37. Devi YS, Seibold AM, Shehu A, Maizels E, Halperin J, Le J, et al. Inhibition of MAPK by prolactin signaling through the short form of its receptor in the ovary and decidua: involvement of a novel phosphatase. J Biol Chem. 2011 Mar;286(9):7609–18.

38. Budd SJ, Aris RM, Medaiyese AA, Tilley SL, Neuringer IP. Increased plasma mannose binding lectin levels are associated with bronchiolitis obliterans after lung transplantation. Respir Res. 2012;13:56.

39. Carroll KE, Dean MM, Heatley SL, Meehan AC, Mifsud NA, Kotsimbos TC, et al. High levels of mannose-binding lectin are associated with poor outcomes after lung transplantation. Transplantation. 2011 May;91(9):1044–9.

40. Munster JM, van der Bij W, Breukink MB, van der Steege G, Zuurman MW, Hepkema BG, et al. Association between donor MBL promoter haplotype and graft survival and the development of BOS after lung transplantation. Transplantation. 2008 Dec;86(12):1857–63.

41. Takahashi H, Sano H, Chiba H, Kuroki Y. Pulmonary surfactant proteins A and D: innate immune functions and biomarkers for lung diseases. Curr Pharm Des. 2006;12(5):589–598.

42. Kwakkel-van Erp JM, van de Graaf EA, Paantjens AWM, van Ginkel WGJ, Schellekens J, van Kessel DA, et al. The killer immunoglobulin-like receptor (KIR) group A haplotype is associated with bronchiolitis obliterans syndrome after lung transplantation. J Heart Lung Transplant. 2008 Sep;27(9):995–1001.

43. Kastelijn EA, van Moorsel CH, Kazemier KM, Roothaan SM, Ruven HJ, Kwakkel-van Erp JM, et al. A genetic polymorphism in the CAV1 gene associates with the development of bronchiolitis obliterans syndrome after lung transplantation. Fibrogenesis Tissue Repair. 2011;4:24.

44. Tiriveedhi V, Angaswamy N, Weber J, Mohanakumar T. Lipid raft facilitated ligation of K-alpha1-tubulin by specific antibodies on epithelial cells: Role in pathogenesis of chronic rejection following human lung transplantation. Biochem Biophys Res Commun. 2010 Aug;399(2):251–5.

45. Keane MP, Gomperts BN, Weigt S, Xue YY, Burdick MD, Nakamura H, et al. IL-13 is pivotal in the fibro-obliterative process of bronchiolitis obliterans syndrome. J Immunol. 2007 Jan;178(1):511–9.

46. Morrell MR, Pilewski JM, Gries CJ, Pipeling MR, Crespo MM, Ensor CR, et al. De novo donor-specific HLA antibodies are associated with early and high-grade bronchiolitis obliterans syndrome and death after lung transplantation. J Heart Lung Transplant. 2014 Dec;33(12):1288–94.

47. Budding K, van de Graaf EA, Otten HG. Humoral immunity and complement effector mechanisms after lung transplantation. Transpl Immunol. 2014 Oct;31(4):260–5.

48. Safavi S, Robinson DR, Soresi S, Carby M, Smith JD. De novo donor HLA-specific antibodies predict development of bronchiolitis obliterans syndrome after lung transplantation. J Heart Lung Transplant. 2014 Dec;33(12):1273–81.

49. Kugler MC, Joyner AL, Loomis CA, Munger JS. Sonic hedgehog signaling in the lung. From development to disease. Am J Respir Cell Mol Biol. 2015 Jan;52(1):1–13.

50. Lappi-Blanco E, Lehtonen ST, Sormunen R, Merikallio HM, Soini Y, Kaarteenaho RL. Divergence of tight and adherens junction factors in alveolar epithelium in pulmonary fibrosis. Hum Pathol. 2013 May;44(5):895–907.

51. Inghilleri S, Di Carlo S, Morbini P, Calabrese F, Politano G, Zampieri D, et al. System biology (SB) allows the identification of pathogenic micro RNA (miR) in BOS. European Respiratory Journal. 2015;46(suppl 59). Available from: `http://erj.ersjournals.com/content/46/suppl_59/PA1791`.
